# Why caution should be applied when interpreting and promoting findings from Mendelian randomisation studies

**DOI:** 10.1136/gpsych-2023-101047

**Published:** 2023-08-10

**Authors:** Alice R Carter, Abigail Fraser, Laura D Howe, Sian Harris, Amanda Hughes

**Affiliations:** 1 MRC Integrative Epidemiology Unit, University of Bristol, Bristol, UK; 2 Population Health Sciences, University of Bristol, Bristol, UK

**Keywords:** Biological Psychiatry, Mendelian Randomization Analysis, Mental Health

## Introduction

In their article entitled ‘Investigating genetic causal relationships between blood pressure and anxiety, depressive symptoms, neuroticism and subjective well-being’, Cai and colleagues^1^ presented the results of a two-sample Mendelian randomisation^2^ (MR) study examining associations between blood pressure traits (systolic, diastolic, hypertension and pulse pressure) and psychological traits (anxiety, depression, neuroticism and subjective well-being). After correction for multiple testing, the authors reported a small but statistically significant result for the effect of diastolic blood pressure (DBP) on neuroticism (an increase of 0.0036 standard deviation (SD) per mm Hg increase in DBP), which they argued demonstrated a causal relationship. All other results were null. However, key aspects of their approach mean that caution is warranted regarding their conclusion. Here we attempt to replicate their results, highlighting key issues with how these methods have been implemented. We also consider the wider limitations of MR approaches and discuss the need for caution when interpreting MR results.

Summary data-based MR (often also referred to as two-sample MR) uses summary statistics from genome-wide association studies (GWAS) to estimate the association between genetic variants and exposures of interest on the one hand, and between the same genetic variants and outcomes on the other, to explore whether associations between exposures and outcomes are likely to reflect a causal effect. Because genetic variants are determined before birth and do not change during a person’s lifetime, MR studies are less impacted by classical confounding and reverse causation than non-genetic observational studies. An overview of MR methods and their assumptions is given in online supplemental box 1.

10.1136/gpsych-2023-101047.supp1 Supplementary data

Crucially, Cai *et al* described their analysis as a bidirectional MR study: one that investigated two possible causal directions of an association and compared the evidence for each. However, the authors only carried out analysis for the impact of blood pressure traits on psychological outcomes, not the converse. This has important implications for the interpretation of results, which are presented as supporting a causal effect of DBP on neuroticism but not vice versa. Interestingly, the authors did not examine the reverse causal path, deciding to run analyses only where 10 or more genetic variants were independently associated with the exposure with an F-statistic of >10. This is an unusual decision given that methods exist for MR using a single genetic variant^3^ (we apply these later in this commentary).

Cai and colleagues applied generalised summary data-based MR (GSMR)^4^ with genetic associations drawn from both peer-reviewed GWAS and online databases whose contents may have undergone less stringent quality control (see Cai *et al*, table 1). Estimates are calculated using the heterogeneity in dependent instruments (HEIDI) outlier test to minimise the impact of horizontal pleiotropy. This refers to the situation in which the exposure-associated genetic variants are associated with the outcome via pathways independent of the exposure, which can inflate causal effects in MR. Nevertheless, they rely on a single method to infer causality rather than presenting estimates from a range of MR methods, each with different assumptions and limitations.

**Table 1:** Number of SNPs and F-statistics for exposures

Phenotype	Clumping threshold (R^2^)	Maximum number of SNPs associated with exposure at p<5×10^−8^*	Mean F-statistic across all SNPs
Diastolic blood pressure	<0.05	1145	60.42
	<0.001	455	79.42
Systolic blood pressure	<0.05	1063	59.11
	<0.001	456	75.13
Pulse pressure	<0.05	1445	59.13
	<0.001	484	81.79
Hypertension	<0.05	88	46.00
	<0.001	72	47.97
Anxiety	<0.001	0	NA
Depressive symptoms	<0.001	2	38.75
Neuroticism	<0.001	10	38.45
Subjective well-being	<0.001	1	27.56

*For some analyses, not all these SNPs were used. This was where not all the SNPs were available in, or could not be harmonised with, the outcome GWAS. For the number of SNPs used in each analysis, see online supplemental table 1.

GWAS, genome-wide association study; NA, not available; SNP, single nucleotide polymorphism.

An absence of horizontal pleiotropy is one of the three core assumptions of MR; however, there are several further sources of bias relevant to MR studies.^2^ In Cai *et al*’s study, the sample populations used to derive associations of genetic variants with exposures and outcomes contain many of the same participants. Sample overlap between the exposure and outcome GWAS can lead to overfitting bias in MR, where MR estimates are exaggerated away from the null.^5^ Although recent methodological work suggests bias due to sample overlap is minimal when strong instruments are used (ie, F-statistics >10), this potential source of bias should still be acknowledged where overlapping samples are used, particularly where methods are available to estimate the size of and correct for any bias.^6^ This is especially pertinent when a single large study (eg, UK Biobank) contributes heavily to both the exposure and outcome GWAS.

In the GWAS used by Cai *et al*, blood pressure was adjusted for body mass index (BMI) to increase statistical power. However, the adjustment for a covariate can bias the GWAS results and subsequent MR estimates that are based on the covariate-adjusted GWAS estimates.^7^ Moreover, for MR, genetic instruments should be strongly associated with the exposure (a genome-wide significant p value threshold of <5×10^–8^) and independent of one another. Typically, independent instruments are identified using a linkage disequilibrium (LD) clumping threshold of R^2^<0.001, but Cai *et al* used an unusually liberal threshold of R^2^<0.05. This can overestimate the precision of results, leading to type 1 errors (false positive results). A further technical challenge is the use of binary exposure variables formed by dichotomising an underlying continuous distribution which can violate^8^ a key assumption of MR. For example, hypertension is used as an exposure; however, hypertension is a dichotomisation of two continuous variables: systolic blood pressure (SBP) and DBP.

While the authors do consider pleiotropy as a non-causal explanation for associations, they do not acknowledge or discuss the potential impact of demographic or family-level processes^9^ that can bias MR estimates based on genetic associations calculated in samples of unrelated individuals.^10^ Lastly, the authors draw causal conclusions from results based on a single MR method. MR approaches are imperfect, and, for this reason, conclusions are more robust when based on results from several methods considered together,^11^ as recommended in the MR-Strengthening the Reporting of Observational Studies in Epidemiology guidelines.^12^ If GSMR supports much stronger causal claims than other genetic approaches when used on its own, the authors do not clarify why this would be so.

## Replication: methods

Here we attempt to replicate Cai *et al*’s analyses. Unfortunately, code was not provided to allow identical replication. Increasingly, it is considered standard practice for researchers to share code used in the analysis,^13^ and not doing so may compound limitations of original work by making reproducibility harder. Using the same sources as Cai *et al* means some of the same issues will impact our replication : adjustment for BMI in blood pressure GWAS, overlap of sample populations used to derive exposure and outcome associations and bias due to family-level processes. However, it allows a clearer exploration of how other decisions by the authors impact their results. For three of the four psychological traits (depressive symptoms, neuroticism, subjective well-being (SWB)), we investigate both causal directions: the impact of blood pressure on psychological traits and the impact of psychological traits on blood pressure (this is not possible for anxiety, with which no genetic variants are associated at the conventional significance threshold (p<5×10^–8^)). Where possible, we apply several widely used and complementary MR methods: the inverse variance-weighted, weighted mode and MR-Egger methods. Where there are too few independent genetic associations to apply these methods, we calculate Wald ratios. For the impact of blood pressure traits on neuroticism, we explore how the threshold used to identify independent genetic associations with blood pressure affects results. We use an LD clumping threshold of R^2^<0.001 and a distance of 10 000 kb (incidentally, the default settings provided in the TwoSampleMR R package^14^). In all analyses, we had suitable instrument strength (mean F>10), which notably was higher for psychological traits when a more stringent LD clumping threshold was used (table 1) than that applied by Cai *et al*. Using the same liberal LD threshold as Cai *et al*, we had a similar number of single nucleotide polymorphisms (SNPs) for analysis of blood pressure traits as the exposure.

Analyses were conducted using R V.4.2.2. We used the TwoSampleMR package, which includes harmonisation of exposure and outcome SNPs, using default parameters. For graphs, we used Stata V.17. We have made all analysis codes available at https://github.com/alicerosecarter/BP_PsychologicalTraits_MR_Replication to allow reproducibility and replication of our analyses.

## Replication: results

**Figure 1:**
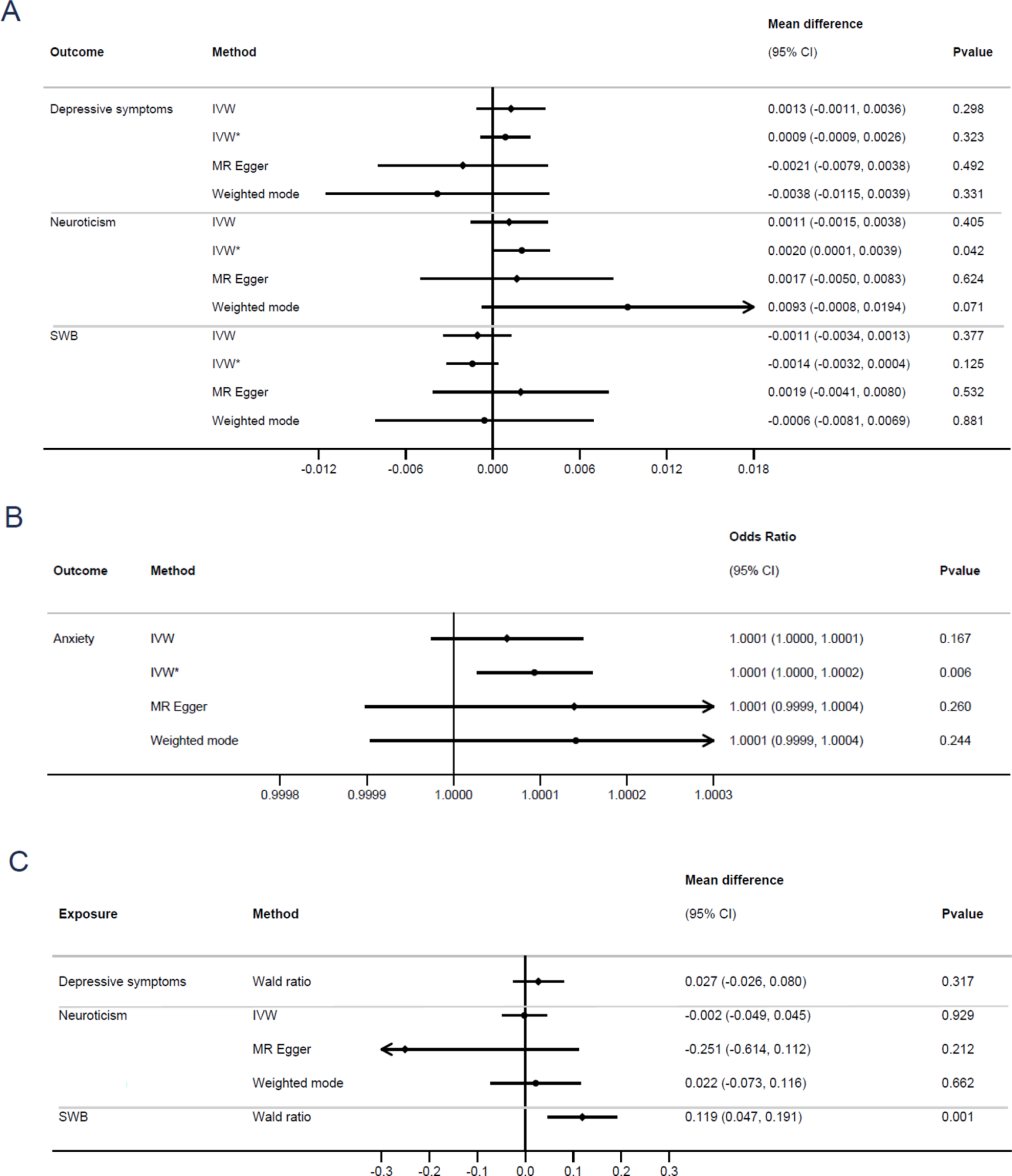
The bidirectional association between DBP and anxiety, depressive symptoms, neuroticism and SWB. (A) Results with DBP as the exposure for continuous outcomes on the mean difference scale. (B) Results with DBP as the exposure for dichotomous outcomes on the odds ratio scale. (C) Results with DBP as the outcome on the mean difference scale. P values have not been corrected for multiple testing. CI,confidence interval; DBP, diastolic blood pressure; IVW, inverse variance-weighted method, LD clumping threshold of <0.001; IVW*, inverse variance-weighted method, LD clumping threshold of <0.05 as used by Cai *et al*; LD, linkage disequilibrium; MR, Mendelian randomisation; SWB, subjective well-being.

Our results provide little evidence that any blood pressure trait affects any psychological trait (figure 1, online supplemental figures 1–3). Cai *et al* reported a 0.0036 SD increase in neuroticism (SE: 0.0008) per mm Hg increase in genetically instrumented DBP. Our primary analysis found an equivalent association of 0.001 SD (SE: 0.0014). We were unable to replicate the authors’ support for the impact of DBP on neuroticism using inverse variance-weighted, weighted mode and MR-Egger methods with a conventional threshold (R^2^<0.001) for independent genetic instruments for the exposure. However, applying the same liberal threshold as in the original paper (R^2^<0.05) resulted in evidence of an effect of DBP on neuroticism using the inverse variance-weighted method, although this would not survive correction for multiple testing (p=0.042). For example, a stringent Bonferroni-corrected p value threshold would be 0.05/32=0.002 (where 32 is the number of exposure–outcome combinations considered).

In the second half of our bidirectional analysis, psychological traits were treated as the exposures and blood pressure traits as the outcome. Here, results of the Wald ratio method suggested an impact of lower SWB on increasing SBP and hypertension (eg, mean difference in mm Hg of SBP per unit increase in SWB=7.87 mm Hg; p<0.001). However, only a single SNP was available for SWB, and sensitivity analyses using alternative MR methods could not be conducted. We therefore urge against strong causal interpretations based on this result. Should a larger GWAS of SWB be conducted, further analyses to see if this estimate remains robust to conventional MR assumptions may become possible.

## Replication: interpretation

Our analysis, which applied multiple MR methods to examine the impact of blood pressure traits on psychological outcomes and vice versa, finds little evidence for an impact of blood pressure on any psychological outcome. The association reported by Cai *et al* and presented as evidence of a causal impact of DBP on neuroticism could only be replicated with one of four approaches applied and only when using a liberal threshold to identify SNPs from the GWAS to use in analyses—an approach likely to underestimate SEs. This suggests that the previously reported result should be regarded cautiously, as it may be a type 1 error (false positive). On the other hand, our results are consistent with a causal impact of SWB on both hypertension and DBP. However, we were only able to apply one method (the Wald ratio method), which limits the strength of conclusions that can be drawn.

Our analysis has important strengths relative to the original analysis: its application of multiple MR methods, use of more stringent thresholds for identifying independent instruments and bidirectional analysis. At the same time, other limitations of Cai *et al*’s study apply equally to our results because we used genetic associations from the same sources. Besides those noted by Cai and colleagues (residual effects of pleiotropy and limited generalisability beyond European populations), this includes three limitations that were not discussed in the original paper: the overlap of sample populations between exposure and outcome GWAS, the impact of using a blood pressure GWAS that was adjusted for BMI and the possibility that GWAS captures heritable genetic effects and intergenerational non-genetic ones.

## Interpretation of causal effects

Considering the strong causal claims made by Cai *et al*, we were surprised by how little discussion was given to possible biological mechanisms. Instead, they discussed how psychological traits could affect blood pressure (the causal direction which their analyses did not test). Such mechanisms include sympathetic overactivity and autonomic dysfunction, blood pressure-elevating effects of medications used to treat anxiety and other coping strategies (eg, smoking and alcohol intake). The biological mechanisms leading to a potential causal effect of blood pressure on psychological traits are perhaps less clear.

## MR in the media

Cai *et al*’s article received widespread media coverage. As of 20 July 2023, Altmetric had detected 98 news stories about this paper from 87 outlets, with accounts of this research published in French, German, Croatian and Spanish, as well as English. The article has been cited in mainstream newspapers, lifestyle publications and specialist publications aimed at clinicians.

The strength of MR studies is that they are less vulnerable to the types of confounding and reverse causality bias which affect non-genetic studies, but they also have limitations which means strong causal conclusions are not always warranted. This can be difficult to convey to non-specialist audiences, but doing so is crucial. Media reports of the study repeated the claim that DBP has a causal effect on neuroticism and that actions to control blood pressure could reduce neuroticism and self-criticism. Of course, controlling blood pressure has other benefits, and such advice is perhaps unlikely to be harmful. But this may not always be the case.

For this reason, it is crucial that MR studies clearly acknowledge and responsibly communicate the nuance of their findings and their limitations. It is also important that researchers and press teams work together to communicate not only the findings of studies but also their limitations and potential sources of false positives and other errors when deciding whether or not to produce a press release or its specific wording. This is particularly important in communicating health-related information, for in addition to the public-facing coverage, articles in specialist magazines and blogs are important tools to provide updates to busy clinicians.
