# Supplementary material for: Why caution should be applied when interpreting and promoting findings from Mendelian randomisation studies
Source: Gen Psychiatr. 2023 Aug 10;36(4):e101047. doi: 10.1136/gpsych-2023-101047 (PMC10423826; doi:10.1136/gpsych-2023-101047)

Box 1: Overview of Mendelian randomization (MR) assumptions and methods[2]

Main assumptions of MR

MR has three core assumptions:

1. Relevance assumption: The genetic variant(s) are strongly associated with the exposure of interest.
2. Independence assumption: There are no (unmeasured) confounders between the genetic variants and outcomes of interest.
3. Exclusion restriction criteria (no pleiotropy): There is no pathway between the genetic variant(s) and the outcome other than via the exposure of interest.

Additional, more nuanced assumptions apply to the interpretation of MR analyses. This includes that genetic variants act equally in all individuals (instrument homogeneity), and that genetic variants are truly randomly allocated across the population. This is not always true (e.g., due to ancestral clustering of genetic variants). Consideration should be given to core and advanced assumptions before making strong causal claims.

Common statistical approaches to MR

In the case of summary data MR with multiple genetic variants, typically the “main” MR effect will be estimated with a fixed or random-effects inverse variance weighted method. This combines Wald ratios for individual SNP-outcome effects, giving an overall MR estimate. This method is statistically powerful but can be biased in the presence of pleiotropy.

Pleiotropy robust methods: methods have been developed to account for pleiotropic SNPs, including MR-Egger, the weighted median and weighted mode estimators, MR-PRESSO and MR-RAPs. These make different assumptions about the pleiotropic effects of the genetic variants. A combination of these methods, each giving consistent estimates, allows the greatest confidence that results do not only reflect pleiotropy.

Multivariable MR: this allows the joint effects of two or more exposure to be estimated simultaneously. Multivariable MR can be used, for example, to account for a known pleiotropic pathway or to explore mediated effects.

MR is a rapidly evolving field, with new methodological approaches frequently becoming available. Since all have slightly different assumptions, data requirements, strengths and weaknesses, the most appropriate strategy is the application of multiple methods to test the robustness of findings to these differences.

A more extensive introduction to MR assumptions and methods can be found here.

BMJ Publishing Group Limited (BMJ) disclaims all liability and responsibility arising from any reliance placed on this supplemental material which has been supplied by the author(s)

Supplementary Table 1: Number of SNPs and mean F statistics for each individual analysis for A) blood pressure traits as exposures and B) blood pressure traits as outcomes

Supplementary Table 1a: Number of SNPs and mean F statistics for each individual analysis for blood pressure traits as exposures

**Exposure**	**Clumping threshold (r^2)**	**Outcome**							
		**Anxiety**		**Depressive symptoms**		**Neuroticism**		**Subjective wellbeing**	
		Number of SNPs used in analysis	Mean F statistic	Number of SNPs used in analysis	Mean F statistic	Number of SNPs used in analysis	Mean F statistic	Number of SNPs used in analysis	Mean F statistic
**Diastolic blood pressure**	<0.05	631	63.61	1041	60.94	1041	60.94	751	61.66
	<0.001	291	80.01	433	80.05	433	80.05	336	81.24
**Systolic blood pressure**	<0.05	620	60.26	978	59.58	978	59.58	710	60.45
	<0.001	293	74.38	431	75.16	431	75.16	326	76.66
**Pulse pressure**	<0.05	533	59.82	975	59.70	975	59.67	748	60.41
	<0.001	212	79.94	356	82.28	355	82.38	292	81.55
**Hypertension**	<0.05	53	48.52	81	46.61	81	46.61	53	48.72
	<0.001	48	49.93	66	48.66	66	48.66	48	50.77

Supplementary Table 1b: Number of SNPs and mean F statistics for each individual analysis for blood pressure traits as outcomes

**Exposure**	**Outcome**							
	**Diastolic blood pressure**		**Systolic blood pressure**		**Pulse pressure**		**Hypertension**	
	Number of SNPs used in analysis	Mean F statistic	Number of SNPs used in analysis	Mean F statistic	Number of SNPs used in analysis	Mean F statistic	Number of SNPs used in analysis	Mean F statistic
**Anxiety**	0	NA	0	NA	0	NA	0	NA
**Depressive symptoms**	1	38.45	1	38.45	1	38.45	1	38.45
**Neuroticism**	10	38.45	10	38.45	10	38.45	10	38.45
**Subjective wellbeing**	1	27.56	1	27.56	1	27.56	1	27.56

Supplementary Figure 1: The bidirectional association between systolic blood pressure (SBP) and anxiety, depressive symptoms, neuroticism and subjective wellbeing. Panel A presents results with SBP as the exposure for continuous outcomes, panel B presents results with SBP as the exposure for dichotomous outcomes, and panel C with SBP as the outcome.

Footnote: IVW = inverse variance weighted method

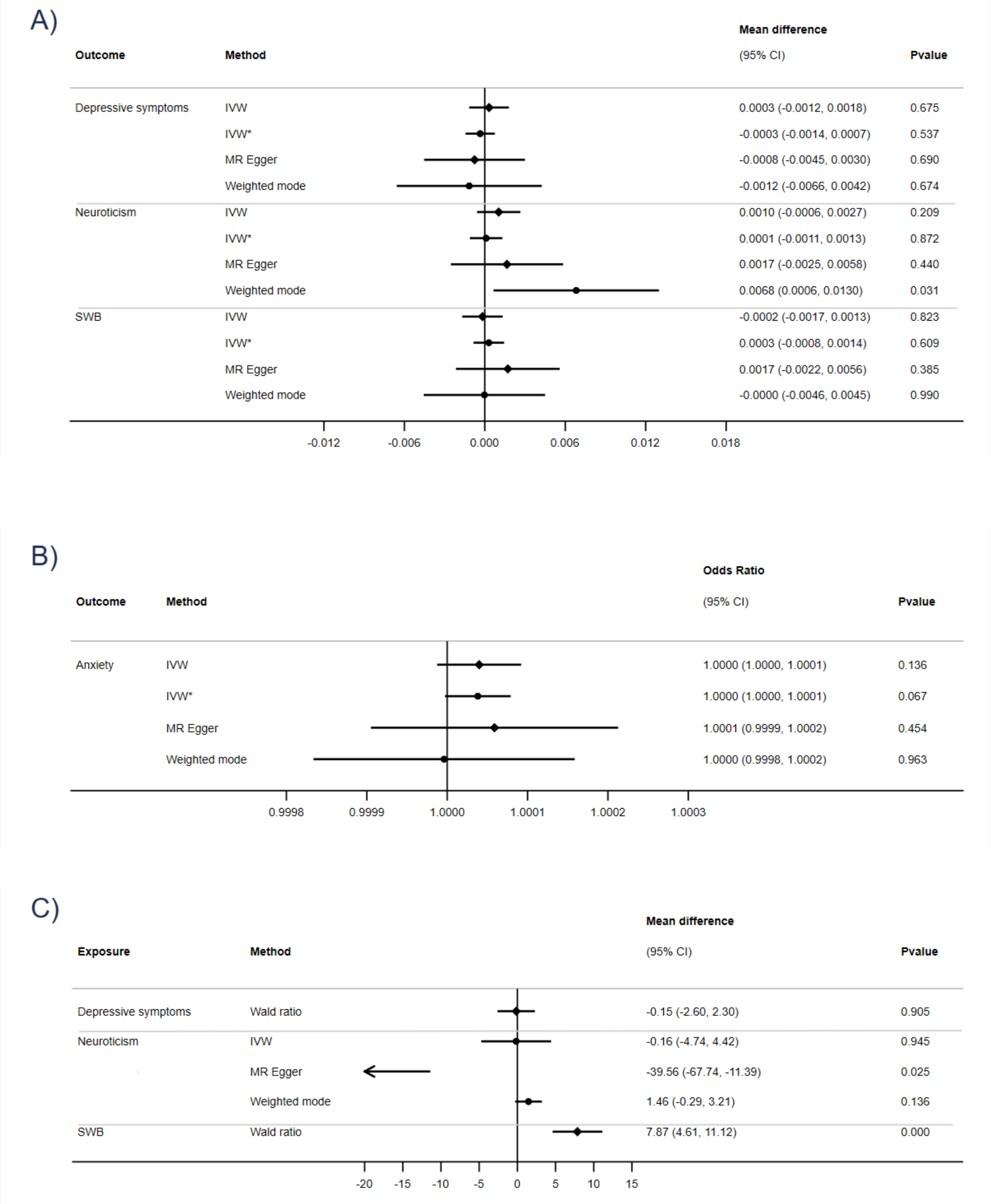

Supplementary Figure 2: The bidirectional association between pulse pressure (PP) and anxiety, depressive symptoms, neuroticism and subjective wellbeing. Panel A presents results with PP as the exposure for continuous outcomes, panel B presents results with PP as the exposure for dichotomous outcomes, and panel C with PP as the outcome.

Footnote: IVW = inverse variance weighted method

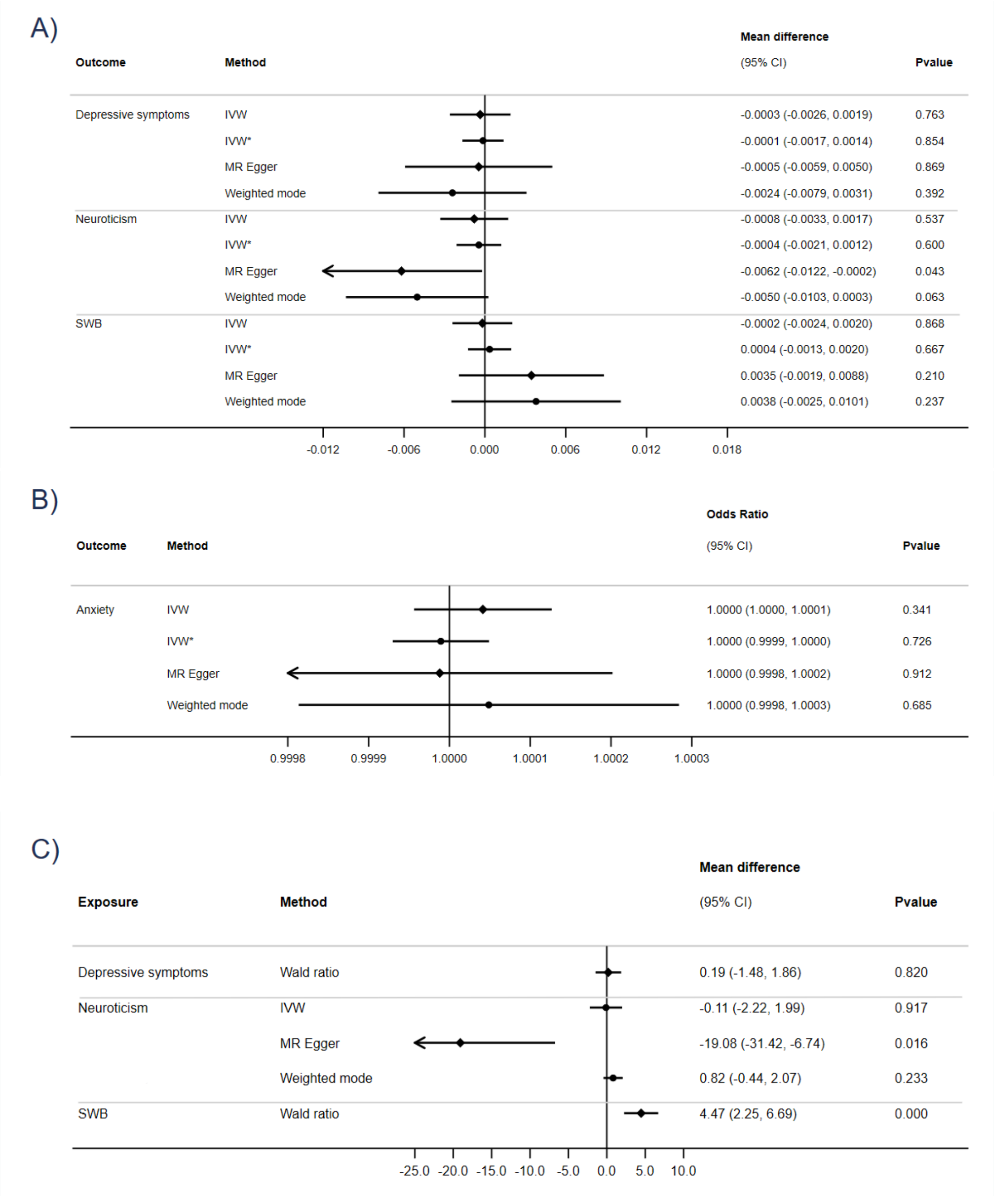

Supplementary Figure 3: The bidirectional association between hypertension and anxiety, depressive symptoms, neuroticism and subjective wellbeing. Panel A presents results with hypertension as the exposure for continuous outcomes, panel B presents results with hypertension as the exposure for dichotomous outcomes, and panel C with hypertension as the outcome.

Footnote: IVW = inverse variance weighted method

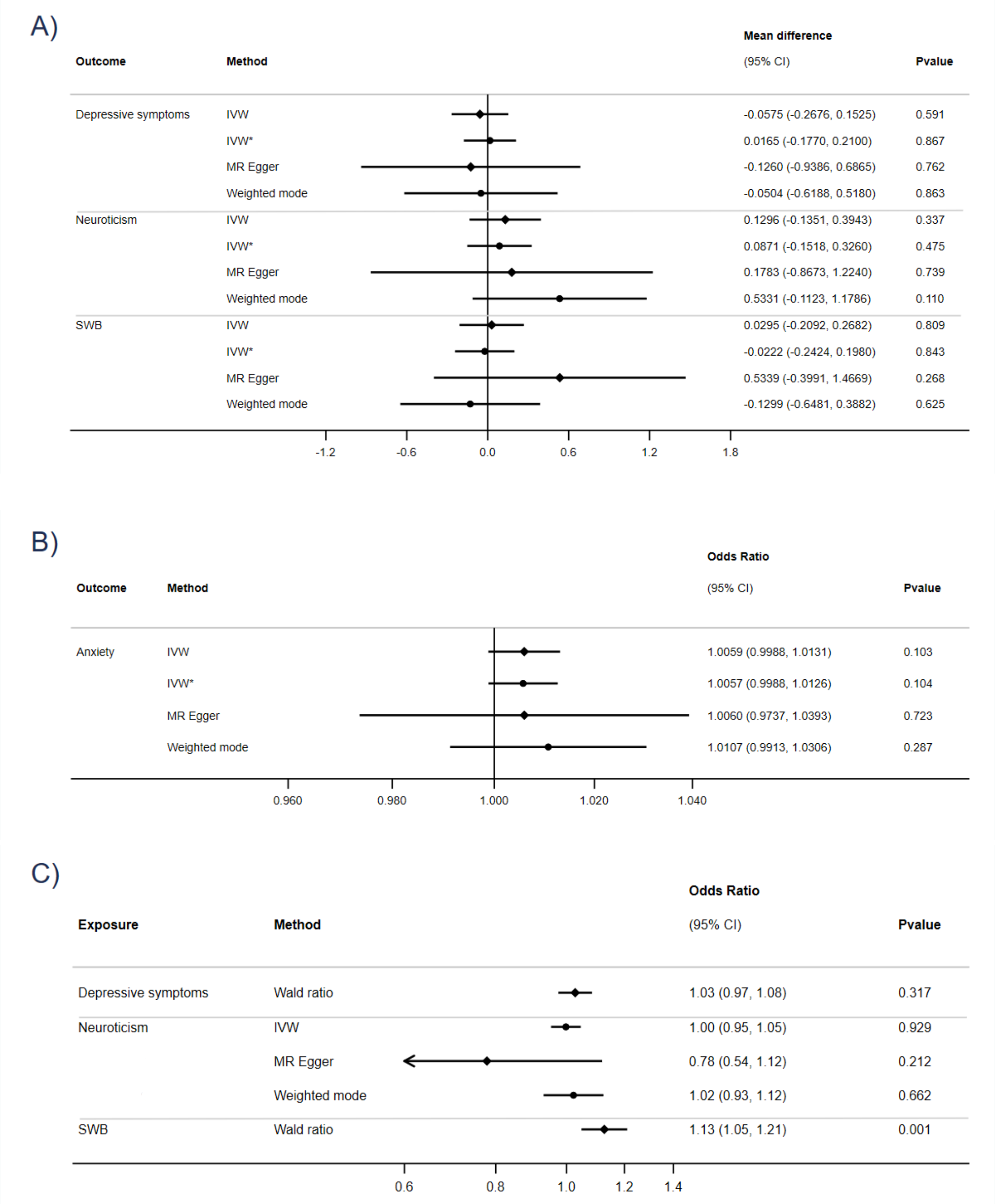

Supplement: Supplementary data [file gpsych-2023-101047supp001.pdf]
